# Acute metabolic decompensation after liver transplant in a patient with maple syrup urine disease

**DOI:** 10.1002/jmd2.12460

**Published:** 2024-11-25

**Authors:** Shao Ching Tu, Marium Khan, Katie Wolfe, Sakil S. Kulkarni, Elizabeth Toolan, Dorothy K. Grange

**Affiliations:** ^1^ Division of Genetics and Genomic Medicine, Department of Pediatrics Washington University School of Medicine St. Louis Missouri USA; ^2^ Division of Pediatric Critical Care Medicine, Department of Pediatrics Washington University School of Medicine St. Louis Missouri USA; ^3^ Division of Gastroenterology, Hepatology and Nutrition, Department of Pediatrics Washington University School of Medicine St. Louis Missouri USA

**Keywords:** decompensation, dehydration, encephalopathy, hyperleucinosis, liver transplant, MSUD

## Abstract

Maple syrup urine disease (MSUD) is an inborn error of metabolism characterized by the accumulation of branched‐chain amino acids (leucine, isoleucine, and valine) caused by a defect in the branched‐chain alpha‐keto acid dehydrogenase complex. Liver transplant is an effective therapy for MSUD, and patients can usually tolerate a regular diet after transplant without symptomatic metabolic decompensation. Most post‐transplant patients do not follow a sick‐day diet. We report a case of a 7‐year‐old male with MSUD Type IA, status post‐liver transplant at 2 years of age, who presented with profound encephalopathy following poor oral intake and vomiting for 3 days. Broad laboratory workup was significant for hyperleucinosis and an unrevealing infectious workup. We conducted a review of eight post‐liver transplant MSUD patients followed at Washington University in St. Louis. The review revealed that plasma amino acids were generally not checked during intercurrent illnesses in this patient cohort. While most of our patients have not had documented encephalopathy, one of the patients with epilepsy had a seizure during a gastrointestinal illness. Based on the review of the literature and from our center's experience, acute metabolic decompensation with intercurrent illnesses in MSUD patients after liver transplant appears to be rare. This case report raises awareness that patients with MSUD are still at risk of developing metabolic crisis post‐liver transplant and provides additional insight into the risk factors associated with metabolic decompensation in this patient cohort.

**Synopsis**

In the form of a case report and a review of patients with maple syrup urine disease, this article highlights the risk of developing metabolic decompensation even after a liver transplant.

## 1 BACKGROUND

Maple syrup urine disease (OMIM #248600) (MSUD) is an autosomal recessive disorder characterized by the accumulation of branched‐chain amino acids (BCAA; leucine, isoleucine, and valine). It is an inborn error of metabolism caused by pathogenic variants in the branched‐chain alpha‐keto acid dehydrogenase complex (BCKD) encoded by *BCKDHA* (OMIM #608348), *BCKDHB* (OMIM #248611), *DBT* (OMIM #248610), and *DLD* (OMIM #238331). These genes form the catalytic subunits of BCKD, which metabolizes BCAA. The clinical presentation of MSUD depends on the residual BCKD activity. The liver accounts for approximately 10% of the total body BCKD activity.^1^, ^2^, ^3^, ^4^ After a liver transplant, the majority of MSUD patients tolerate a normal diet and do not experience symptomatic metabolic crises.^5^, ^6^, ^7^ These observations suggested that liver transplant sufficiently corrects BCAA metabolism. However, there have been 11 reported post‐liver transplant MSUD patients who had hyperleucinosis and/or encephalopathy during intercurrent illnesses.^5^, ^8^, ^10^, ^11^ We report another pediatric patient who had an acute metabolic decompensation during a gastrointestinal illness 6 years following liver transplant. This case report provides additional insight into the risk factors associated with metabolic decompensation in this patient cohort. Additionally, we conducted a retrospective chart review of other post‐liver transplant patients with MSUD, who are followed at our institution. The review showed that although metabolic decompensation during intercurrent illnesses is rare, it may be under‐reported.

### 1.1 Case report

A 7‐year‐old male with MSUD Type IA received a segment two–three cadaveric liver transplant at 2 years of age. Following the transplant, he tolerated an unrestricted diet without any symptomatic metabolic decompensations. Prior to the transplant, he had a history of gross motor delay. Post‐transplant, he caught up on all age‐appropriate milestones and was enrolled in a regular class with no concerns about his school performance. Five years post‐liver transplant, he presented with profound encephalopathy due to hyperleucinosis, in the setting of 3 days of vomiting and decreased oral intake.

Three days prior to admission to our hospital, he presented to a local emergency department (ED) with nausea and vomiting. A review of systems was positive for decreased urine output. There, the initial laboratory workup was unremarkable. He was diagnosed with acute gastroenteritis and was discharged home. His symptoms persisted over the next 2 days. He was eating very little solid food and drinking small amounts of water, Pedialyte, or Prime hydration drink, which reportedly contains 250 mg of BCAAs per bottle. His mental status had reportedly remained normal during this time. However, on the day of admission, he was somnolent with slurred speech and an abnormal gait which prompted re‐evaluation at a local ED and subsequent transfer to the Pediatric Intensive Care Unit (PICU) at our hospital.

At the local ED, initial blood work demonstrated leukopenia with lymphopenia (WBC 4.48 k/μL, absolute lymphocyte count 630/μL, absolute neutrophil count 3240/μL), hypoglycemia (glucose 53 mg/dL), and a high anion gap metabolic acidosis (pH 7.17, bicarbonate 9.5 mmol/L, and anion gap of 14.5 mmol/L). Blood cultures were obtained, and broad‐spectrum empiric antimicrobials were initiated prior to transfer. Upon admission to our PICU, additional blood work showed persistent metabolic acidosis (pH 7.29, bicarbonate 16 mmol/L), hyponatremia (128 mmol/L), and normal transaminases. Additionally, a computed tomography scan of the head revealed no acute intracranial process. Further history revealed that he had recently participated in mushroom scavenging, and examination revealed multiple ticks in his genital region. The differential for his acute encephalopathy remained broad at this point and included an acute metabolic process, an infectious process (including tick‐borne illnesses), and acute dehydration. Thus, all exogenous protein sources were stopped. He was administered 10% dextrose‐containing intravenous (IV) fluids at a 1.5 times maintenance rate and empiric doxycycline was added to his antibiotic regimen while awaiting results of a broad infectious workup (including urine, blood, and cerebrospinal fluid cultures) and additional metabolic testing. Once the PICU team secured a central line, the dextrose‐containing fluids were further concentrated to 20% dextrose‐containing fluids, and he was started on IV SMOF (soybean, MCT, olive oil, and fish oil) lipids at 2 gm/kg.

Plasma amino acid analysis revealed elevated leucine 1885 mcmol/L (reference range: 40–150 mcmol/L), valine 1125 mcmol/L (reference range: 85–320 mcmol/L), and allo‐isoleucine 164 μmol/L (reference range: 15–75 mcmol/L) and the aforementioned broad infectious workup remained negative. Plasma leucine levels rapidly decreased to a normal level within a day of administering continuous IV dextrose fluid (providing a glucose infusion rate ~9 mg/kg/min), with a concurrent significant improvement in his mental status, indicating that the hyperleucinosis was the primary driver of his acute encephalopathy. All antibiotics except doxycycline were discontinued after 24 h of negative blood and urine cultures, and reassuring CSF studies. Due to the presence of ticks on initial exam, a 7‐day course of doxycycline was completed. When he was clinically stable enough to resume enteral feeds, a leucine‐free formula containing l‐valine and l‐isoleucine was initiated continuously via a nasogastric tube. He resumed a regular diet on day 3 of admission and was discharged home on day 4.

After the liver transplant, he remained adherent to immunosuppression with documented therapeutic levels and regular follow‐ups with his transplant team. His transplant complications included a history of atrophy of the right lobe of the liver (segment IVb) 2 months post‐transplant, mild late acute cellular rejection 1‐year post‐transplant, and hepatic vein stenosis requiring angioplasty 5 years post‐transplant. The most recent liver biopsy was performed 5 years post‐transplant, which was about 1 year prior to this admission and demonstrated mild periportal inflammation, features of bile duct injury, and increased portal and periportal fibrosis with thin septa and focal bridging (stage II), with no evidence of acute cellular rejection. With regard to the history of hepatic vein stenosis, hepatic venogram and pressure measurements demonstrated focal stenosis at the hepatic vein anastomosis with a significant venocaval gradient of 16 mmHg. Following angioplasty, the pressure gradient significantly improved down to 4 mmHg. Shortly after discharge following the metabolic decompensation, a venogram of the liver was obtained and demonstrated an unchanged pressure gradient across the hepatic vein and IVC anastomosis without evidence of clinically significant stenosis.

## 2 DISCUSSION

The liver expresses 9%–13% of the body's total BCKD complex activity.^1^, ^5^ Liver transplant is an effective therapy for MSUD, and most patients can tolerate a regular diet after transplant without symptomatic metabolic decompensation. In humans, it has been estimated that the brain expresses 10%–20% and the kidneys each express 8%–13% of the whole‐body BCAA metabolism.^1^ Therefore, BCAA levels often remain mildly elevated in patients, even after liver transplant. There have been a handful of reported cases of hyperleucinosis in transplanted MSUD patients during intercurrent illnesses. Notably, gastrointestinal illnesses were a common presentation among these reports (Table 1).^5^, ^8^, ^10^, ^11^ Based on our review of the literature and our own cases, there is not a specific genotype in classic MSUD that is more susceptible than others to develop metabolic decompensation post‐liver transplant.

**TABLE 1:** Reports of hyperleucinosis in patients with maple syrup urine disease post‐liver transplant.

Patient# [Reference]	E	1 [8]	2 [8]	3 [8]	4 [8]	5 [8]	6 [5]	7 [10]	8 [11]
Age at diagnosis	Newborn								
Age at transplant	2 yo	1 yo	3 yo	2 yo	2 yo	14 months	2 yo	1 yo	1 yo
Donor type	D, seg 2–3	D	LNR	LR (M)	LNR	D	D	LR (M)	LR (F)
Type of infections	GE	GE, RTI	GE, RTI	GE, RTI	N/A	GE	GE	GE	RTI
Max Leu post‐transplant (mcmol/L)	1885	2088	1672	2784	546	1930	2170	2001	450
Home illness management		CHO	MF	CHO	CHO	CHO			
Inpatient management									NA
IV dextrose	Y	Y	Y	Y	Y	Y	Y	Y	
Medical formula	Y		Y	Y				Y	
IV intralipid	Y			Y					

*Note*: Patient “E” is our case report patient. Patients 1–8 are patients who were reported in the literature.

Abbreviations: CHO, carbohydrate; D, deceased; do, day‐old; F, father; GE, gastroenteritis; IV, intravenous; LNR, living non‐related; LR, living related; M, mother; MF, medical formula; RTI, respiratory tract infection; Y, yes; yo, year‐old.

*Source*: Adapted from Guilder et al.^8^

Our center has experience caring for patients with MSUD following their liver transplants. The reported case is the first documented patient from our institution who presented with hyperleucinosis following a liver transplant. After his presentation, we conducted a retrospective chart review on a total of eight transplanted patients with MSUD followed at Washington University in St. Louis. As summarized in Table 2, two of our patients (patients B and D) recovered from hypovolemia and/or multiple episodes of emesis without significant metabolic acidosis or presenting with encephalopathy. However, there was a patient with epilepsy (patient H) who was admitted to an outside hospital with nausea, vomiting, and seizures. The details of his hospital admission were unclear due to limited access to the other facility's medical records, but he was noted to have received symptomatic management and was discharged in a week. Plasma amino acids were not obtained during the intercurrent illnesses to confirm the BCAA levels.

**TABLE 2:** Post‐transplant patients with maple syrup urine disease followed by the Genetics team at Washington University in St. Louis.

	Genotype	Current age	Age at transplant	Transplant type	Post‐transplant complications	Post‐transplant metabolic crises/age at the time	ED visits or hospitalizations
A	Homozygous BCKDHA c.1312T>A, p.Tyr438Asn	18 yo	12 yo	FLC	Stage 2 CKD noted in 2023	NR	None reported
B	N/A	24 yo	19 yo	FLC	Anastomotic biliary stricture, s/p stenting and acute rejection ×1 requiring steroid course‐ now resolved; stage 2 CKD	NR	Admission for hypovolemic shock w/large volume bloody diarrhea, coffee ground emesis POD 36 on 10/11/2019
C	BCKDHB c.503G>A, p.Arg168His; c.509G>A, p.Arg170His	3 yo	5 months	FLC	None (as of 4/28/23)	NR	Admitted for gastrocutaneous fistula closure
D	Homozygous BCKDHA c.1312T>A p.Tyr438Asn (aka Tyr393Asn)	7 yo	3 yo	FLC	None (as of 5/22/24)	NR	ED visit for NBNB emesis ×4, found to have cystitis
E	BCKDHA c.979G>A p.Glu327Lys; c.1312T>A p.Tyr438Asn	8 yo	2 yo	Segment 2–3 cadaveric	Hepatic vein stenosis s/p angioplasty, atrophy of right liver lobe, mild late ACR 2018 ×1 (resolved 2019)	Y/7	Admission for AMS found to have hyperleucinosis
F	N/A	26 yo	9 yo	FLC	Multiple cellular rejections req. augmentation of immunosuppressants; last episode on 3/11/21	NR	None reported
G	N/A	23 yo	11 yo	FLC	AKI, ACR ×1 (6 months post‐op), autoimmune‐mediated hepatitis (2018)	NR	Episodes of emesis that were managed at home
H	N/A	32 yo	16 yo	N/A	Plasma cell rejection ×2, immune mediated hepatitis (2022)	NR	N/V/D/seizures (at 30 yo), CMP with transaminitis (ALT 521, AST 279), without metabolic acidosis (bicarb 26 mmol/L)

Abbreviations: ACR, acute cellular rejection; AMS, altered mental status; ED, emergency department; do, day‐old; FLC, full liver cadaveric; N/V/D, nausea/vomiting/diarrhea; NR, none reported; Y, yes; yo, year‐old.

A catabolic state resulting from poor caloric intake and recurrent emesis certainly could contribute to metabolic derangement. Additionally, our patient had a history of hepatic vein stenosis, status post‐dilation. His most recent IR venogram redemonstrated a discrepancy in the size of the anastomosis at the transplanted hepatic vein and the patient's inferior vena cava. While the anastomosis size does not appear to cause clinically significant stenosis during the patient's normal hydration status, the narrowing may have theoretically rendered our patient more sensitive to dehydration due to decreased orthotopic liver outflow.

Our patient's leucine level returned to normal with 22 h of high dextrose‐containing fluids (glucose infusion rate: 9.4 mg/kg/min) and 8 h of intralipid infusion. Within this timeframe, a total of 1722.8 mL (79 mL/kg) of dextrose‐containing fluids and caloric intake that equates to 49 kcal/kg/day were infused. The caloric intake is below the recommended daily caloric need of 60–65 kcal/kg/day, while the fluid volume exceeded the daily requirement.^9^ Although both catabolism and dehydration likely played a role in the development of metabolic crisis, fluid replenishment likely played a more significant role in our patient's speedy clinical recovery.

In summary, a patient with MSUD suffered from encephalopathy and hyperleucinosis in the setting of poor caloric intake and vomiting for 3 days despite a prior liver transplant. We speculate that our patient's hepatic vein stenosis may have compounded the effect of dehydration, by decreasing the orthotopic liver outflow. In other words, the blood that underwent BCAA degradation by the BCKD of the orthotopic liver required more time to flow into the IVC and thus to the rest of the body. On review of the literature and from our center's experience, acute metabolic decompensation with intercurrent illnesses in MSUD patients after liver transplant appears to be rare. Nonetheless, the families, patients, and medical providers should be informed about the risks of hyperleucinosis during an intercurrent illness even after liver transplantation. The current management strategy for our patients is to check serum amino acids during illnesses and to have a low threshold to start dextrose‐containing IV fluids, particularly if there is concern for dehydration. We do not routinely check serum amino acids, except once a year during follow‐up visits. Given the rarity of these presentations and the increased prevalence of liver transplants in patients with MSUD, a multi‐center evidence‐based consensus on management is needed to minimize metabolic complications post‐transplant.

## AUTHOR CONTRIBUTIONS

Dr. Shao Ching Tu drafted the initial manuscript, collected clinical data, carried out the initial analysis, critically reviewed and revised the manuscript, and was responsible for the overall integrity of the content of the manuscript. Dr. Dorothy K. Grange, Dr. Sakil Kulkarni, and Dr. Katie Wolfe were directly involved in the patient's care and contributed to the manuscript through critical review and revision of the manuscript. Dr. Marium Khan was responsible for obtaining informed consent from the guardian, was directly involved in the patient's care, and contributed to the manuscript by critical review and revision of the manuscript.

Elizabeth Toolan, MS, RD, LD was directly involved with the patient's care and made substantial contributions to the data acquisition by organizing a list of patients with MSUD status post‐liver transplant followed at Washington University in St. Louis. She also contributed to the manuscript by a critical review of the manuscript. All the authors approved the final manuscript as submitted.

## CONFLICT OF INTEREST STATEMENT

The authors declare no conflicts of interest or financial disclosures.

## INFORMED CONSENT

Informed consent was obtained from the guardian for the publication of anonymized data. No identifying patient information is presented in the manuscript.
